# The Impact of Food Viscosity on Eating Rate, Subjective Appetite, Glycemic Response and Gastric Emptying Rate

**DOI:** 10.1371/journal.pone.0067482

**Published:** 2013-06-20

**Authors:** Yong Zhu, Walter H. Hsu, James H. Hollis

**Affiliations:** 1 Department of Food Science and Human Nutrition, Iowa State University, Ames, Iowa, United States of America; 2 Department of Biomedical Sciences, Iowa State University, Ames, Iowa, United States of America; University of Warwick – Medical School, United Kingdom

## Abstract

Understanding the impact of rheological properties of food on postprandial appetite and glycemic response helps to design novel functional products. It has been shown that solid foods have a stronger satiating effect than their liquid equivalent. However, whether a subtle change in viscosity of a semi-solid food would have a similar effect on appetite is unknown. Fifteen healthy males participated in the randomized cross-over study. Each participant consumed a 1690 kJ portion of a standard viscosity (SV) and a high viscosity (HV) semi-solid meal with 1000 mg acetaminophen in two separate sessions. At regular intervals during the three hours following the meal, subjective appetite ratings were measured and blood samples collected. The plasma samples were assayed for insulin, glucose-dependent insulinotropic peptide (GIP), glucose and acetaminophen. After three hours, the participants were provided with an *ad libitum* pasta meal. Compared with the SV meal, HV was consumed at a slower eating rate (P = 0.020), with postprandial hunger and desire to eat being lower (P = 0.019 and P<0.001 respectively) while fullness was higher (P<0.001). In addition, consuming the HV resulted in lower plasma concentration of GIP (P<0.001), higher plasma concentration of glucose (P<0.001) and delayed gastric emptying as revealed by the acetaminophen absorption test (P<0.001). However, there was no effect of food viscosity on insulin or food intake at the subsequent meal. In conclusion, increasing the viscosity of a semi-solid food modulates glycemic response and suppresses postprandial satiety, although the effect may be short-lived. A slower eating rate and a delayed gastric emptying rate can partly explain for the stronger satiating properties of high viscous semi-solid foods.

## Introduction

Food rheology is the branch of science that deals with the flow and deformation of foods. While being important to consumer acceptance of food, a growing body of evidence indicates that the rheological properties of food, such as physical form, contribute to altered appetite and modulate glycemic response. For example, several studies have reported that fluid calories, such as beverages, are less satiating than their solid equivalents [1], [2], [3], [4] and induce a larger rebound fall in postprandial glucose concentrations [5], [6]. These effects on appetite and glycemic response could further modify the risk of chronic diseases such as obesity [7] and diabetes [8]. However, less is known about whether more subtle differences in food rheological properties, for example, differences in the viscosity of a semi-solid food, would have a similar effect on appetite and glycemic response. Increasing current knowledge on this topic may aid the development of new or reformulated functional food products for health promotion [9], [10].

Several studies using foods in different forms have found that increasing the viscosity of a food reduces food intake [11], [12] or suppresses appetite [13], [14]. Nonetheless, comparing different food forms, such as a beverage vs a semi-solid or solid food, may not give a true indication of the effect of viscosity on appetite due to cognitive differences in how participants view the test products (e.g., liquid beverages quench thirst whereas semi-solid or solid foods sate hunger) [15]. In addition, the physiological mechanisms that explain these observations have received little attention. Recently, it was reported that standardizing eating rate of a liquid meal and a semi-solid meal resulted in no difference in *ad libitum* food intake; however, the intake was significantly different if eating rate was not controlled [12]. While accumulating evidences suggest the association of eating rate and appetite [16], [17], the effect of food viscosity on eating rate warrants further investigation. Increasing food viscosity may also delay gastric emptying rate [13], [18], [19], although conflicting results have also been reported [20]. The delayed gastric emptying may prolong satiety, as gastric distention is a key influence on feelings of fullness [21], [22].

In addition to an effect on appetite, food viscosity may also modulate postprandial glycemic and insulin response, as the delay in the gastro-intestinal transition of viscous meals would likely slow the rate of digestion and absorption. Nonetheless, current studies provide inconsistent results related to the effect of meal viscosity on postprandial glucose and insulin response [19], [20], [23], [24], [25] and further research is warranted to clarify this matter. In addition, little is currently known about the effect of food viscosity on the response of glucose-dependent insulinotropic peptide (GIP), a hormone secreted in response to digestion in the small intestine to facilitate disposal of ingested nutrients and stimulate insulin secretion [26].

The objective of the present study was to investigate the effect of viscosity of a semi-solid food on eating rate, subjective appetite, glycemic response, hormones related to glucose metabolism, as well as gastric emptying rate. Our hypothesis was that increasing food viscosity would reduce subjective appetite, due to a slower eating rate and delayed gastric emptying rate. We also hypothesized a viscous meal would result in a lower GIP and insulin response, together with a blunted post-prandial glycemic response.

## Materials and Methods

### Participants

Participants were recruited for this study using an email sent to faculty, staff and students at Iowa State University and flyers posted in the local community. Participants interested in the study attended a screening session to determine their eligibility. At this screening session the participants were asked to complete a questionnaire that consists of questions related to their general health, such as self-reported diseases and medication use, as well as questions from the three-factor eating questionnaire [27]. In addition, their height and body weight were measured using a stadiometer and a calibrated clinical weighing scale. From these measures, body mass index was calculated. Potential participants were also asked to taste each of the foods used in the study and rate their palatability on a 9 point scale. Participants were eligible for the study if they: were male, aged between 18–40 years, were of self-reported good health, and had a BMI between 20.0 and 29.9 kg/m^2^. Participants were excluded from the study if they: used tobacco products, had the presence or history of gastrointestinal disease, had the presence of acute or chronic disease, had diagnosed eating disorder, were a restrained eater (>13 on the restraint section of the three-factor eating questionnaire [27]), were using medication or rated the palatability of any of the test foods lower than 5 on a 9-point scale [28], [29]. This study was approved by the Iowa State University Institutional Review Board and all participants signed an informed consent form before being included in the study.

### Test Meal

The standard viscosity meal (SV) consisted of 318 g chocolate pudding (Kozy Shack Inc, Hicksville, NY, USA), 30 g heavy whipping cream (Anderson Erickson Dairy, Des Moines, IA, USA) and 1000 mg acetaminophen that was used as a marker of gastric emptying rate [30]. The high viscosity meal (HV) was made using the same ingredients as the SV but with the addition of 3.3 g guar gum (Frontier Natural Products, Norway, IA, USA). All meals were prepared using standard procedures and the ingredients were mixed thoroughly. The nutrient composition of the meals was determined using nutrient analysis software (Nutritionist Pro, version 4.6, Axxya Systems, Stafford, TX, USA), which reported that the test meal provided 1690 kJ (404 kcal) energy with 12% of total energy from protein, 57% from carbohydrate and 31% from fat. The food was served at 4°C.

**Figure 1:**
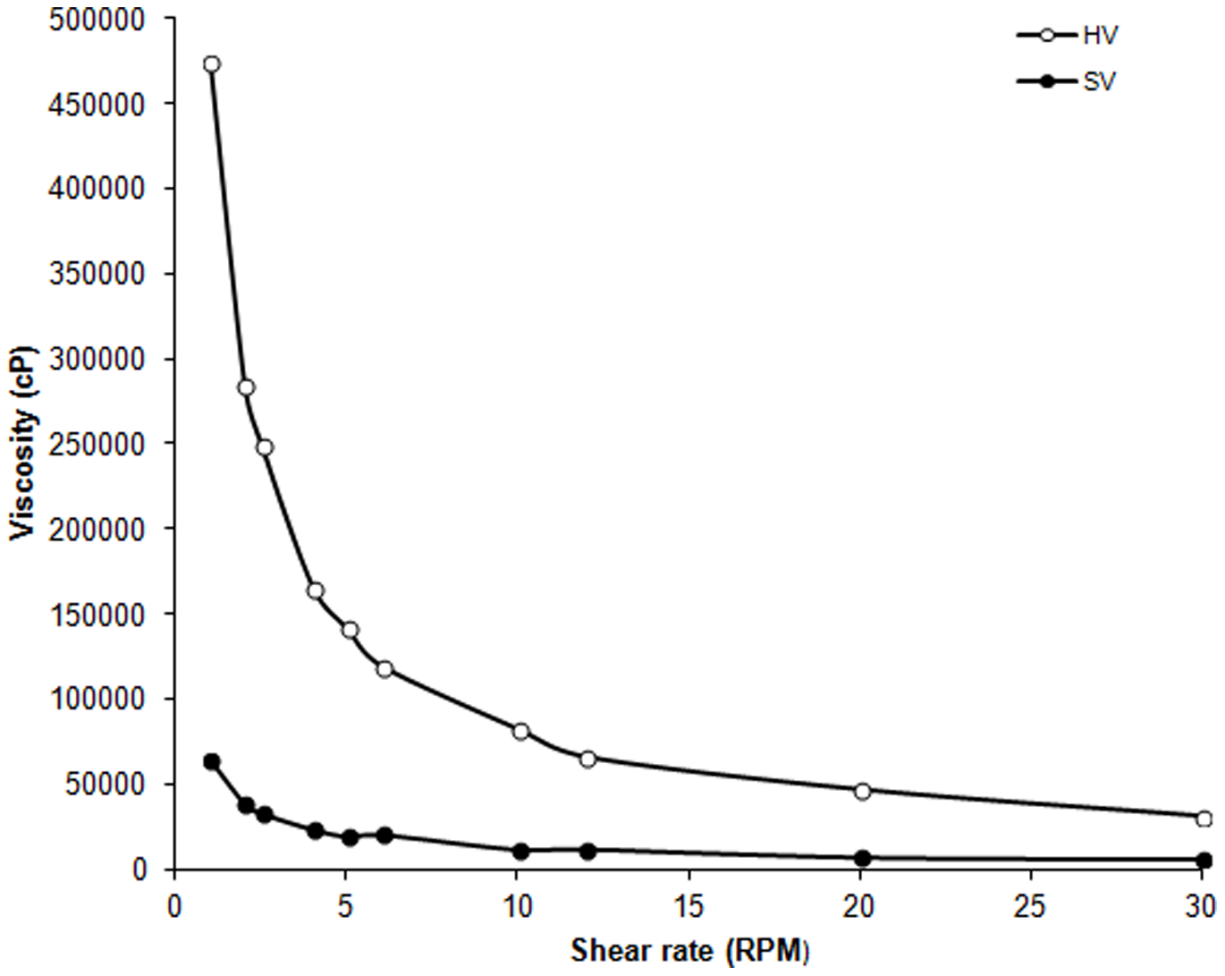
The viscosity of the standard viscosity meal (SV) and the high viscosity meal (HV).

Both meals were in a semi-solid form. The viscosity of the test foods was measured using a DV-I prime viscometer (Brookfield Engineering, Middleboro, MA, USA) with spindle # 6, using a 250 mL beaker at room temperature (Figure 1). Comparison of palatability ratings revealed there was no difference in the palatability of the test meals (P = 0.11).

Pasta meals were prepared in 3766 kJ (900 kcal) portions, made by 150 g Barilla spaghetti (Barilla America Inc., Bannockburn, IL, USA), 375 g tomato sauce (Barilla America Inc., Bannockburn, IL, USA), 37.5 g shredded parmesan cheese (Wal-Mart Stores Inc., Bentonville, AR, USA) and 5.1 g salt using a standard cooking protocol. Nutrient analysis revealed 17% of the energy was from protein, 65% from carbohydrate and 18% from fat. This meal was served at 60°C.

### General Procedure

This study used a randomized cross-over design. Participants attended two test sessions that were separated by a 7-day washout period. Participants were instructed to avoid alcohol consumption and strenuous physical activity for the 48 hours before each test session. On each test day, they were required to report to the laboratory at 7∶30 am following an overnight fast of at least 12 hours. After reporting to the laboratory, an indwelling catheter was inserted into their non-dominant arm. Following a thirty-minute acclimatization period, a baseline blood sample was taken and a baseline appetite questionnaire completed. The appetite questionnaire posed four questions: how hungry do you feel right now? How full do you feel right now? How preoccupied with food are you right now? What is your desire to eat right now? Responses were captured using a 100 mm visual analogue scale (VAS). The VAS was anchored with diametrically opposed statements (e.g. not at all hungry, as hungry as I have ever felt). Immediately following the baseline measurement the participant was presented with the relevant test food (SV or HV) and asked to consume the meal in its entirety. The time taken to eat the meal was measured to assess the eating rate using a stopwatch. The participant was not aware that eating rate was being measured until after the study was completed. Immediately after finishing the meal another blood sample was taken (t0). Further blood draws were made at t0+15, 30, 45, 60, 90, 120 and 180 minutes. At each time point participants also completed a fresh appetite questionnaire. Throughout the test session, participants were required to remain seated in a quiet room that was free from food cues. After the final blood draw, the indwelling catheter was removed and participants were allowed to rest for five minutes before being presented with the pasta meal. Participants were instructed to eat until comfortably full and they were informed that an extra portion was available if they needed. Each bowl of food was weighed before and after serving out of sight of participants. During the study period, participants were isolated from each other using screens. In addition, water was not allowed to be consumed as it could confound our measurements on appetite and gastric emptying rate.

### Gastric Emptying Rate Measurement

Gastric emptying rate was assessed using the acetaminophen absorption test [30]. For this assay, blood was drawn into lithium heparin coated vacutainer tubes at each time point and centrifuged at 3000 g at 4°C for 15 minutes. Plasma acetaminophen concentration was assayed by HPLC using the method described by Jensen *et al.* [31]. The mobile phase consists of 0.1 M potassium dihydrogen orthophosphate, isopropanol and tetrahydrofuran (v/v/v: 100∶1.5∶0.1, pH 3.7).

### Glucose, Insulin and GIP Measurement

These assays were conducted using the same protocol as described in an earlier study [32]. Briefly, blood was drawn into 4 mL EDTA coated vacutainer tubes and mixed with 400 µL 10000 KIU/ml aprotinin and centrifuged at 3000 g at 4°C for 15 minutes. The plasma was divided into aliquots and stored at −80°C until analysis. Glucose was analyzed by a biochemical analyzer (YSI Life Sciences, Model 2700 select, Yellow Springs, OH, USA). Radioimmunoassay was used to analyze concentrations of insulin and GIP. For insulin assay, the intra-assay CV was 8% and the inter-assay CV was 8% at 20 µU/mL. The intra-assay CV for GIP assay was 3% and its inter-assay CV was 5% at 0.5 ng/mL.

### Statistical Analysis

All data are presented as mean ± standard error of the mean. A sample size calculation indicated that 15 participants would provide 80% power to detect a difference of 8% in outcome measures at the significance level of 0.05. SAS v9.2 (SAS Institute, Cary, NC, USA) was used to perform statistical analysis. A mixed model repeated measures ANOVA (Proc Mixed, SAS) was used to test the overall treatment effect, time effect and treatment×time interaction on subjective appetite, hormones and metabolites measured from blood samples. Baseline values were included as a covariate whereas participants were added as a random variable in the model. Post-hoc analysis was performed using a Bonferroni adjusted pairwise comparison of responses from the same time point. Differences in meal duration, eating rate, and food intake at the *ad libitum* meal was tested using a paired t-test.

## Results

### Participant Characteristics, Meal Duration and Eating Rate

Participants (n = 15) had a mean age of 27±2 years (range: 19–37) and a mean BMI of 24.2±0.5 kg/m^2^ (range: 21.4–28.0 kg/m^2^). Meal duration was significantly shorter for the SV meal (SV 234±20 seconds vs HV 391±51 seconds, P = 0.008). The overall eating rate for two meals, calculated by dividing weight of food by meal duration, was significantly different (HV 1.19±0.18 g/s vs SV 1.74±1.16 g/s, P = 0.020).

### Subjective Appetite

**Figure 2:**
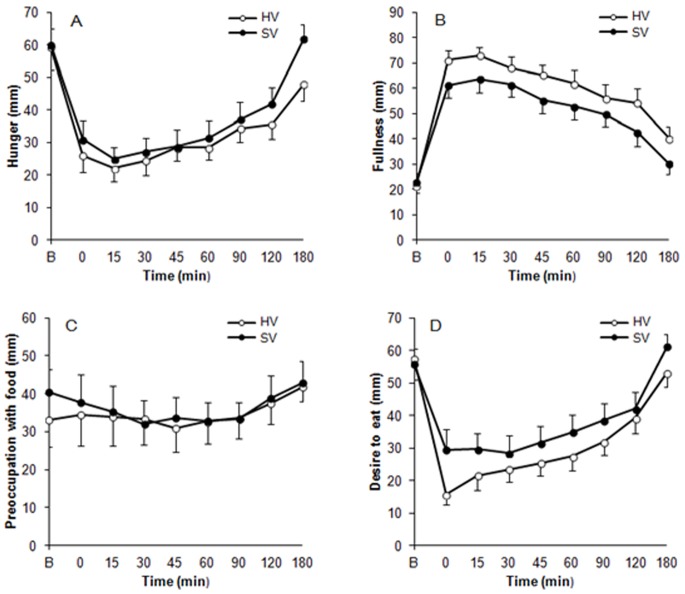
Hunger (A), fullness (B), preoccupation with food (C) and desire to eat (D) after consuming a standard viscosity meal (SV) and a high viscosity meal (HV). Main effect of treatment was significant on hunger, fullness and desire to eat (P = 0.019, P<0.001, P<0.001 respectively).

Figure 2 illustrates the subjective appetite response to SV and HV. A significant main effect of time was found for all parameters (P<0.001) except preoccupation with food (P = 0.202). There were no statistically significant treatment by time interactions for any of these parameters (P>0.05).

There was a significant main effect of viscosity on hunger (P = 0.019, Figure 2A). Compared with SV, hunger was lower when HV was consumed. The main effect of viscosity on fullness was significant (P<0.001, Figure 2B) with post-prandial fullness being higher following consumption of HV. There was no significant main effect of viscosity on preoccupation with food (P = 0.739, Figure 2C) but it was significant on desire to eat (P<0.001, Figure 2D) with desire to eat being lower following consumption of HV.

### Glucose, Insulin and GIP

**Figure 3:**
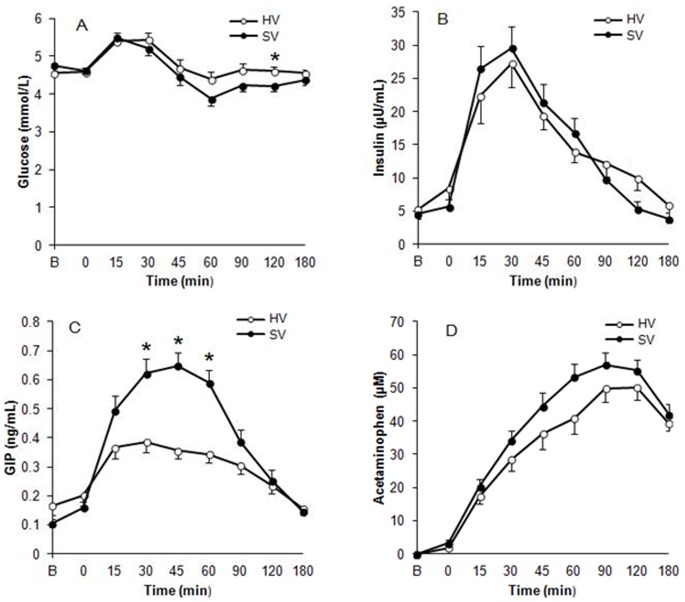
Plasma concentrations of glucose (A), insulin (B), GIP (C) and acetaminophen (D) after consuming a standard viscosity meal (SV) and a high viscosity meal (HV). Main effect of treatment was significant on glucose (P<0.001), GIP (P<0.001) and acetaminophen (P<0.001). * indicates a significant difference between treatment conditions at the same time point.

Figure 3A–C illustrates the plasma concentration of glucose, insulin, GIP after consuming SV and HV. There was a significant main effect of time on all those parameters (P<0.05).

Consuming HV resulted in higher plasma glucose concentrations compared with SV (P<0.001). Post-hoc comparison indicates plasma glucose was significantly higher at 120 min following consumption of HV (P = 0.034). When SV was consumed, plasma glucose peaked at 15 minutes, while it occurred at 30 min following the HV meal.

There was no significant main effect of viscosity on plasma insulin concentrations (P = 0.490). However, a significant main effect of viscosity on GIP was found, with post-prandial GIP concentrations being higher after SV (P<0.001). In addition, treatment by time interactions were significant on GIP (P<0.001). Post-hoc analysis revealed that plasma GIP concentration was higher at 30 min, 45 min and 60 min following consumption of SV (P<0.0001 for all).

### Gastric Emptying Rate

The main effect of food viscosity on plasma acetaminophen concentrations was significant (P<0.001, Figure 3D). The plasma acetaminophen concentration was lower following consumption of HV, indicating the gastric emptying rate was slower following consumption of HV.

### Food Intake

No participant requested additional pasta at the subsequent meal consumed three hours later. There was no difference in food intake at this *ad libitum* meal (HV 493.5±51.8 g vs SV 494.5±50.0 g, P = 0.971).

## Discussion

The present study found that increasing the viscosity of a semi-solid food resulted in a slower eating rate, reduced postprandial hunger and desire to eat, and increased fullness. Increasing food viscosity also resulted in a slower gastric emptying rate and a lower post-prandial plasma GIP concentration. The postprandial plasma concentration of glucose was higher following the HV meal. However, there was no effect on plasma concentration of insulin or food intake at a subsequent meal.

Results from the present study suggest that increasing the viscosity of a semi-solid food has a similar appetite-suppressing effect as reported by those studies using foods in different physical forms [13], [14], [19], [24]. In agreement with studies that measured food intake at the subsequent meal [14], [24], the appetite-suppressing effect was not sufficiently strong to reduce food intake at a meal eaten 3 hours later. The length of time between the test meal and lunch in this present study may have been too long for a relatively small preload to influence energy intake at the lunch meal [33]. Indeed, the one study that found that consuming a viscous meal reduced food intake at the next meal used a shorter duration (90 minutes) between the preload and the test meal [34]. Consequently, it may be interesting to determine if the consumption of a viscous snack between meals is able to reduce food intake at the subsequent meal or aid weight management in people who habitually consume snack foods. For individuals who do not consume snacks, it is unlikely that they would accrue any benefit for weight management by adding a snack to the diet and increasing eating frequency [35], [36]. It is interesting to note that participants were less hungry, had a lower desire to eat and a higher fullness immediately prior to the lunch meal yet this had no effect on food intake. It is possible that the observed differences in appetite were not sufficiently large to influence food intake and were overwhelmed by other drivers of food intake. For example, we provided an excess of free food which may have stimulated food intake for reasons other than satisfying appetite [37]. Another potential explanation is that appetite and food intake are not tightly coupled in the short-term [38] and repeated exposures to a food may be required before a consistent appetitive response is observed. Further studies that examine the influence of food viscosity on appetite and food intake over a longer term are required to identify the link.

It has been suggested that a slower eating rate may contribute to suppressed appetite [5], [16], [17], [39]. While results for our study shows HV resulted in a slower eating rate and reduced appetite, the difference in meal duration we observed was substantially shorter (less than four minutes) than those eating rate studies where the difference was up to 25 minutes [5], [16], [17], [39]. It seems unlikely that such a small difference in meal duration could influence satiety over several hours. Nonetheless, as sensory exposure depends on the exposure duration and the intensity of the stimulus, a slower eating rate would prolong the oral exposure time, which can increase the overall exposure to sensory stimulation in oral and retro-nasal cavity, resulting in suppressed appetite or reduced food intake [40], [41], [42].

The present study found that increasing in food viscosity slows gastric emptying which is consistent with results from most studies that have investigate the impact of viscosity on gastric emptying [13], [18], [19]. This reduction in gastric emptying rate may contribute to satiety by prolonging gastric distention [22]. However, it should be noted that the degree to which food viscosity influences gastric emptying might be markedly reduced by rapid dilution by gastric juice or increased motor function [43], [44]. This is illustrated by a study by Marciani *et al.* [43] who reported that when participants consumed meals that varied 1000-fold in viscosity, gastric emptying rate only differed by a factor of 1.3. Further research is required to determine how food characteristics influence food breakdown in the gastrointestinal tract.

While there was no effect of food viscosity on insulin, we found that consuming the HV meal resulted in a significantly lower GIP response with a higher postprandial plasma glucose concentration with a later occurrence of the glucose peak. There is still not a consistent picture regarding the role of food viscosity on the glycemic response with studies suggesting that higher viscosity meals result in a lower glycemia response [19], [23], [25] no difference [24] or a higher glycemia response [20]. These discrepant results may be due to differences in the methods used to increase the viscosity of the food or differences in the characteristics of participants (e.g., diabeteic or non-diabetic). With regards to guar gum, the dose added to the food may also alter the glycemic response. Torsdottir *et al.* [45] have shown that while low or medium amounts of guar gum reduced plasma concentrations of glucose, a higher amount resulted in higher plasma glucose concentrations [45].

In this study we did not measure appetite-related hormones, which is a limitation. A similar investigation was conducted and it was found that the viscosity of a meal does not influence plasma concentrations of hormones related to appetite in healthy participants [24]. As suggested by Zijlstra et al. [24], this could be because nutrients that entered the gastro-intestinal tract were the same in different test sessions, leading to the same appetite hormone responses [24]. Other studies have found that increasing the viscosity of a meal does affect plasma concentrations of hormones related to appetite [19], [46]. Several factors may explain these discrepant results. First, these studies used different products to test the effect of viscosity on appetite (e.g., beverages and semi-solid foods) and there may be an interaction between viscosity and the delivery medium. Second, various thickening agents, such as starch [24], oat-bran [19], and beta-glucan [46] were used. As there may be substantial differences in how these different ingredients behave in the gastrointestinal tract (e.g., ability to gel in an acid environment, digestion by enzymes), it is possible that the effect of viscosity on appetite hormones may be due, in part, to the characteristics of the thickening agent. In this study a fixed preload for all participants rather than a meal designed to meet a certain percentage of energy need for each participant was provided. This could potentially be a confounding factor. To investigate this issue, a post-hoc calculation was performed. Using the equations for estimating basal metabolic rate [47], with a physical activity level of 1.5, we estimated the energy requirement for each participant. Results suggest the test meal provided 14.5±0.3% (range 13.1–16.1%) of their energy needs. This minor variation is because the study involved only lean and overweight participants. Consequently, the conclusions from this present study cannot be extended to other populations, such as obese people. The effect of food viscosity on appetite in the obese population warrants further studies.

In conclusion, this present study suggests that the viscosity of a semi-solid food modulates glycemic response and influences postprandial satiety, by altering eating rate and gastric emptying rate. Future work should be conducted to fully elucidate how rheological properties of food affect postprandial metabolism and nutrients’ bioavailability, and how does long-term modification of rheological properties of diet influence the risk of chronic diseases.
